# Dissociating Explicit and Implicit Timing in Parkinson’s Disease Patients: Evidence from Bisection and Foreperiod Tasks

**DOI:** 10.3389/fnhum.2018.00017

**Published:** 2018-02-06

**Authors:** Giovanna Mioni, Mariagrazia Capizzi, Antonino Vallesi, Ángel Correa, Raffaella Di Giacopo, Franca Stablum

**Affiliations:** ^1^Department of General Psychology, University of Padova, Padua, Italy; ^2^Department of Neuroscience, University of Padova, Padua, Italy; ^3^San Camillo Hospital IRCCS, Venice, Italy; ^4^Centro de Investigación Mente, Cerebro y Comportamiento, University of Granada, Granada, Spain; ^5^Departamento de Psicología Experimental, University of Granada, Granada, Spain; ^6^Institute of Neurology, San Bortolo Hospital, Vicenza, Italy; ^7^Center for Mind/Brain Sciences (CIMeC), University of Trento, Trento, Italy

**Keywords:** Parkinson participants, basal ganglia, time bisection task, foreperiod, explicit timing, implicit timing, sequential effects

## Abstract

A consistent body of literature reported that Parkinson’s disease (PD) is marked by severe deficits in temporal processing. However, the exact nature of timing problems in PD patients is still elusive. In particular, what remains unclear is whether the temporal dysfunction observed in PD patients regards explicit and/or implicit timing. Explicit timing tasks require participants to attend to the duration of the stimulus, whereas in implicit timing tasks no explicit instruction to process time is received but time still affects performance. In the present study, we investigated temporal ability in PD by comparing 20 PD participants and 20 control participants in both explicit and implicit timing tasks. Specifically, we used a time bisection task to investigate explicit timing and a foreperiod task for implicit timing. Moreover, this is the first study investigating sequential effects in PD participants. Results showed preserved temporal ability in PD participants in the implicit timing task only (i.e., normal foreperiod and sequential effects). By contrast, PD participants failed in the explicit timing task as they displayed shorter perceived durations and higher variability compared to controls. Overall, the dissociation reported here supports the idea that timing can be differentiated according to whether it is explicitly or implicitly processed, and that PD participants are selectively impaired in the explicit processing of time.

## Introduction

Parkinson’s disease (PD) is a progressive neurodegenerative disease characterized by motor and non-motor disorders, such as bradykinesia, tremor, rigidity, olfactory loss, sleep, behavioral and cognitive impairment (Nalls et al., 2015). This heterogeneous disease involves dysfunctions in several circuits, including the loss of dopaminergic neurons in the substantia nigra pars compacta, which has strong implications for the efficacy of the nigrostriatal dopaminergic pathway (Alberico et al., 2017), the loss of dopaminergic neurons in the ventral tegmental area, which mostly affects the mesocortical pathway to the prefrontal cortex (Parker et al., 2015a; Kim et al., 2017), and the degeneration of the cholinergic system, which helps explain the cognitive symptoms of patients with PD (Calabresi et al., 2006).

A well-known hypothesis in the timing literature is that temporal processing in the milliseconds to seconds range involves the basal ganglia and is modulated by the level of dopamine (Meck et al., 2008; Marinho et al., 2018). Supporting the role of the basal ganglia in timing processes, PD patients usually have severe deficits on various temporal tasks and with various temporal ranges (for a review, see Jones and Jahanshahi, 2014). However, the exact nature of timing problems in PD is still elusive. In a critical review of the literature on time perception, Coull and Nobre (2008) fractionated temporal processing and timing tasks on the basis of whether the underlying mechanisms were explicitly or implicitly engaged. In explicit timing tasks, participants are instructed to attend to the duration of the stimulus, which is hence explicitly task-relevant. Conversely, in implicit timing tasks, no explicit instruction to process time is received, albeit timing is inherent in the task to be performed and usually affects behavior. The main goal of the present study is to delve further into the dissociation between explicit and implicit timing in PD.

The dopaminergic system has been associated with both the perception of time intervals in the supra-seconds range and the regulation of speed of a hypothesized internal clock, which is consistent with its effect on the rate of an internal pacemaker that varies between individuals leading to a “faster” clock for some and a “slower” clock for others (Coull et al., 2012). The involvement of the basal ganglia and the dopaminergic system in explicit timing would thus explain the deficit of PD participants in the most commonly used temporal tasks, such as finger-tapping (Artieda et al., 1992; Pastor et al., 1992b; O’Boyle et al., 1996), time reproduction, time production, and time estimation tasks (Pastor et al., 1992a; Lange et al., 1995; Perbal et al., 2005). However, some studies reported that the temporal deficit associated with PD might be explained by impairment of other cognitive processes, such as memory and attention, rather than by a real “clock problem” (Malapani et al., 1998; Koch et al., 2008). Moreover, most of the previous commonly used temporal tasks included a motor component that might have emphasized the observed temporal impairment (for a review, see Jones and Jahanshahi, 2014). Indeed, when the motor component was reduced by using a time bisection task, mixed results have been observed. In the time bisection task, participants are instructed to categorize the presented duration as being more similar to the short or to the long standard interval. Employing the time bisection task, for instance, Smith et al. (2007) showed lower temporal abilities in PD participants compared to controls when using long (1–5 s) temporal intervals, but not when using short ones (100–500 ms). In the study by Merchant et al. (2008), PD participants displayed higher temporal variability than their controls when presented with brief temporal intervals (350–1000 ms). Wearden et al. (2008) found no evidence of temporal impairment in PD participants within the sub-second range (100–800 ms). Finally, Zhang et al. (2016) showed temporal overestimation and higher variability in PD participants with respect to controls using auditory stimuli (330–750 ms). Overall, these mixed results may be explained by clinical (severity and/or medication state) and methodological differences between the studies such as the specific modality and temporal range used.

A classic example of implicit timing task is given by the foreperiod paradigm, in which participants have to respond to a target stimulus preceded by a warning signal (for reviews, see Niemi and Näätänen, 1981; Coull, 2009; Vallesi, 2010). The foreperiod is the time interval between warning and target. When one foreperiod only (e.g., either a short interval of 1000 ms or a long interval of 3000 ms) is presented during a block of trials, response times (RTs) are usually shorter for the blocks with the shorter foreperiod, a phenomenon dubbed the “fixed foreperiod effect” (e.g., Mattes and Ulrich, 1997; Vallesi et al., 2009). The fixed foreperiod effect has been explained in terms of better time estimation of short intervals relative to long intervals (see Gibbon, 1977), which in turn will lead to shorter RTs in the short foreperiod blocks (e.g., Bausenhart et al., 2008).

Unlike the fixed foreperiod paradigm, when shorter and longer foreperiods are randomly and equiprobably intermixed across trials, the pattern of results usually reverses with shorter RTs for the long foreperiod trials, a phenomenon known as the “variable foreperiod effect” (e.g., Niemi and Näätänen, 1981; Mento et al., 2015). A further phenomenon that emerges in the variable foreperiod paradigm concerns the “sequential effects”. Sequential effects consist of a performance benefit when the current short foreperiod is preceded by another short rather than a longer foreperiod. Performance at the current long foreperiod is instead fast irrespective of whether the previous foreperiod has been shorter than or as long as the current one (e.g., Los and van den Heuvel, 2001; Steinborn et al., 2008; Capizzi et al., 2015; Mento, 2017).

Converging evidence from behavioral (Vallesi et al., 2013, 2014), neuropsychological (Vallesi et al., 2007a; Triviño et al., 2010), developmental (Vallesi and Shallice, 2007) and transcranial magnetic stimulation (Vallesi et al., 2007b) studies suggest that dissociable processes may underlie foreperiod and sequential effects (but see Los et al., 2014, for an alternative model). In particular, it seems that sequential effects are mediated by more automatic processes than those at the basis of the foreperiod effect. From a neural point of view, for instance, while the foreperiod effect has been shown to rely on the functioning of prefrontal structures related to executive processes, this is not the case for sequential effects. Such a neural dissociation has led to hypothesize that sequential effects probably rely upon more primitive brain areas that develop earlier as compared to prefrontal structures (Vallesi and Shallice, 2007; see also Mento and Tarantino, 2015). Among these sub-cortical regions, the basal ganglia might be a likely neural substrate for sequential effects. This expectation, however, was not fulfilled in the study by Triviño et al. (2010), which showed normal sequential effects in participants with basal ganglia lesions. The authors attributed the null finding to the fact that their participants had suffered a unilateral stroke that mainly affected the striatum (putamen and caudate nucleus) while leaving intact the substantia nigra and the dopamine production (see also Triviño et al., 2016). Therefore, the administration of a variable foreperiod task to PD participants is critical to directly investigate the involvement of the basal ganglia in the generation of sequential effects.

As regards the foreperiod effect, early studies on PD hypothesized a reduction of the foreperiod effect for PD participants on the ground that such an effect should also depend on intact dopaminergic pathways (e.g., Zahn et al., 1963; Brown and Robbins, 1991). However, a reduced foreperiod effect in PD has not always been confirmed (e.g., Rafal et al., 1984). For instance, Jurkowski et al. (2005) found that PD participants had a normal foreperiod effect in a reflexive (startle-eyeblink) task but not in a voluntary (hand-grip) one. Their conclusion was that interval processing associated with lower level reflexive behavior was intact in PD participants. Likewise, Lee et al. (2012) tested phasic arousal and temporal preparation. Considerable benefit was indeed observed from the warning stimulus, however, the benefit was not greater for the controls than it was for PD participants.

As far as we know, only a few studies have directly compared the performance of PD participants and control participants in both explicit and implicit timing tasks within a single experimental session. Amongst these, de Hemptinne et al. (2013) employed an oculomotor paradigm, which required anticipation of a salient target that moved along a circular path and reversed direction after a short (1200 ms) or long (2400 ms) forward path. The results showed that the explicit timing of target motion but not the implicit one was impaired in PD participants. Most germane to our study for the kind of tasks employed, Jones et al. (2008) study used time production (30, 60 and 120 s) and time reproduction tasks (250, 500, 1000 and 2000 ms) as measures of explicit timing and warned and unwarned reaction time tasks (250, 500, 1000, 2000 ms fixed between blocks) as measures of implicit timing. In the case of time reproduction and warned and unwarned reaction time tasks, PD participants were as accurate as controls when requiring temporal processing within the 250–2000 ms range. Exploratory factor analysis also suggested that the time production task used mechanisms distinct from those employed in time reproduction and warned and unwarned reaction time tasks. The authors concluded that the integrity of the basal ganglia is necessary for producing time in the seconds range and that explicit and implicit timing are mediated by dissociable mechanisms.

Taken together, the previous studies investigating explicit and implicit timing in the same group of PD participants suggest that these two ways of processing time may be differently affected in PD. This is also supported by functional magnetic resonance imaging (fMRI) studies showing that explicit timing engages the basal ganglia, whereas implicit timing does not (Coull and Nobre, 2008). In the present study, we shall further investigate the performance of PD participants when tested with explicit and implicit timing tasks. We opted for a time bisection task to investigate explicit timing (Mioni et al., 2016, 2017) and a foreperiod task to test implicit timing (Vallesi et al., 2014). The time bisection task has been extensively used to study temporal processing and, importantly, has been previously employed with PD participants because the motor component is limited (Mioni et al., 2016, 2017). The foreperiod task was a simple detection task also with low motor demands, in which participants were required to respond to a target stimulus presented either after a fixed foreperiod or a variable one. The use of a variable foreperiod design allowed us to also analyze sequential effects in PD. In addition to explicit and implicit timing tasks, all participants performed neuropsychological tests that evaluated attention, working memory and executive functions, which are usually reduced in PD (Kudlicka et al., 2011).

To sum up, on the basis of prior studies (de Hemptinne et al., 2013; Jones and Jahanshahi, 2014), we expected a deficit of PD participants in the explicit timing task, but not in the implicit one, which would confirm the distinction between the two time processing in PD. Importantly, our work aimed to also shed new light into another implicit temporal phenomenon, namely, sequential effects, which so far have been neglected in the study of time processing in PD.

## Materials and Methods

### Participants

Twenty right-handed PD participants (11 males, 9 females) and 20 right-handed healthy controls (9 males, 11 females) matched for age (*t*_(38)_ = 0.45; *p* = 0.658; *d* = 0.14) and years of education (*t*_(38)_ = 0.11; *p* = 0.911; *d* = 0.03) were examined (Table 1). The sample size was based on previous literature about explicit and implicit temporal processing in PD participants (Jurkowski et al., 2005; Jones et al., 2008; Lee et al., 2012; Mioni et al., 2016, 2017; Zhang et al., 2016). PD participants were recruited and tested at the Center for Neurocognitive Rehabilitation (CeRiN), Center for Mind/Brain Sciences (CIMec), University of Trento (Italy). Control participants were volunteers from the local community (Trento, Italy). All participants received the PD diagnosis (Diagnosis and Treatment of Parkinson’s disease: Italian Guidelines. Health Care Institute and Italian League for Parkinson’s disease, Extrapyramidal Syndrome and Dementia, 2015) by a movement disorders neurologist. All participants were assessed when in “on” medication. The motor involvement of participants was mild, according to the score of the Unified Parkinson’s disease Rating Scale (UPDRS; Movement Disorder Society Task Force on Rating Scales for Parkinson’s Disease, 2003) Part III (medium score: 17/108 point) and Hohen & Yahr Scale (score ≤ 3; Goetz et al., 2004).

**Table 1:** Descriptive statistics (mean and standard deviation) for controls and Parkinson’s disease (PD) participants; *t* and *d* values are also reported.

	Controls *n* = 20 M (*SD*)	PD participants *n* = 20 M (*SD*)	*t*	*d*
Age	69.90 (9.85)	68.60 (8.51)	0.45	0.14
Education (years)	9.80 (4.45)	9.95 (3.94)	0.11	0.03
MMSE	29.05 (0.77)	27.73 (1.63)	3.18*	1.03
Digit Span forward	5.52 (0.90)	5.65 (0.81)	0.45	0.15
Digit Span backward	4.21 (1.31)	4.05 (1.19)	0.40	0.12
TMT Part A (sec)	46.84 (23.67)	60.40 (25.24)	1.73	0.58
TMT Part B (sec)	124.42 (53.66)	185.23 (84.93)	2.60*	0.86
TMT B–A (sec)	77.58 (47.42)	128.35 (68.78)	2.60*	0.87
Attentional matrices	52.47 (6.00)	44.05 (8.24)	3.63*	1.17
Semantic fluency	41.00 (9.67)	36.70 (11.49)	1.26	0.40
Phonemic fluency	36.63 (7.90)	33.80 (15.68)	0.71	0.23
CPM	31.63 (3.16)	28.30 (5.60)	2.27*	0.73
MCST categories	5.00 (1.29)	4.61 (1.64)	0.80	0.26
MCST errors	5.58 (4.83)	4.11 (4.74)	0.93	0.35
CDT	11.00 (1.63)	11.10 (1.77)	0.18	0.06

Note: MMSE, Mini Mental State Examination; TMT, Trial Making Test; CPM, Colored Raven’s Progressive Matrices; MCST, Modified Card Sorting Test; CDT, Clock Drawing Test. *p < 0.05.

The exclusion criteria included: dementia or severe cognitive impairment (Dubois et al., 2007), medications (apart from PD treatments) known to interfere with cognitive functioning, history of neurosurgery or brain injury, psychiatric disorders, or any condition (e.g., drowsiness) that would interfere with testing. Participants recruited obtained at least a score equal to or greater than 24/30 at the Mini-Mental State Examination (MMSE; Folstein et al., 1975). All participants performed an extensive neuropsychological evaluation to investigate their cognitive abilities (Table 1).

### Materials

#### Explicit Timing: Time Bisection Task

**Figure 1:**
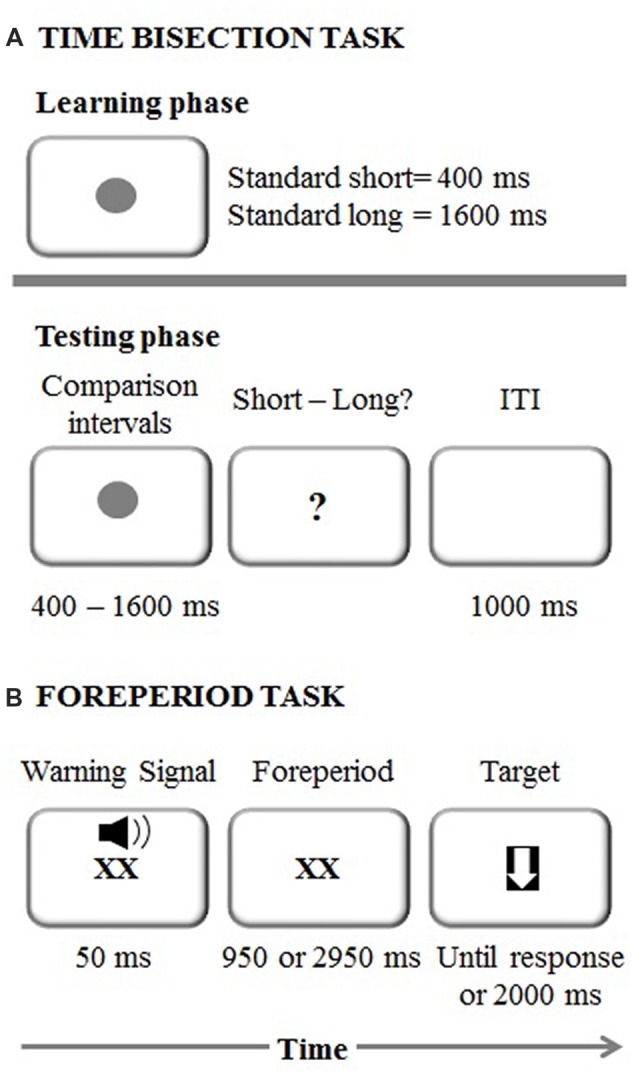
**(A)** Experimental procedure representing the time bisection task. ITI stands for Inter-Trial Interval **(B)** Experimental procedure representing the foreperiod task.

The experimental session started with the learning phase in which each participant memorized two standard durations: 400 ms (short standard) and 1600 ms (long standard; Mioni et al., 2016; Figure 1A). The stimulus used was a dark gray circle on a white background. Both standard durations were presented 10 times in a fixed presentation order. After the learning phase, participants were required to judge the duration of new intervals and determine if they appeared more similar in duration to the short standard or long standard. Seven comparison durations were used: 400, 600, 800, 1000, 1200, 1400 and 1600 ms. Participants performed four blocks and within each block each duration was presented 10 times in a random order. They were asked to respond with their left and right index fingers and response keys were counterbalanced between participants. After each response, there was a 1000-ms inter-trial interval.

#### Implicit Timing: Foreperiod Task

The foreperiod paradigm was a shortened version of the task used in Vallesi et al. (2014; Figure 1B). Each trial started with the presentation of a “XX” (2 cm × 2 cm), which was displayed in the center of the screen simultaneously with an auditory warning signal (a 1500 Hz pure tone) played for 50 ms via laptop internal speakers. The sound intensity was set at a comfortable level for all the participants. The “XX” remained on the screen for either 1000 or 3000 ms, depending on the foreperiod for that trial. The target was a downward-pointing white arrow (with maximum length and width of 2 cm) fitted in a black square, which appeared once the foreperiod duration elapsed. Participants were instructed to respond to it by pressing the spacebar as quickly as possible. Following the response to the target, or after 2000 ms in case of a missed response, the next trial began.

The foreperiod task comprised three types of blocks: fixed-short (1000 ms), fixed-long (3000 ms) and variable (1000 or 3000 ms), which were presented in a counterbalanced order across participants. In total, there were two fixed blocks of 30 trials each (i.e., one block for the short foreperiod and one block for the long foreperiod) and two blocks of 30 trials each for the variable foreperiod. In the variable foreperiod blocks only, the current foreperiod could be preceded with the same probability either by a short or long foreperiod. An initial training phase with four trials was used before each type of block to ensure that participants correctly understood task instructions.

#### Neuropsychological Assessment

A complete neuropsychological evaluation was conducted^1^. Specifically, to assess attention we used the *Digit Span forward and backward* tests (Mondini et al., 2011), the *Trail Making Test* (TMT; Giovagnoli et al., 1996), and the *Attentional Matrices test* (Spinnler and Tognoni, 1987). To assess executive functions we used the *Semantic Fluency test* (Novelli et al., 1986), the *Phonemic Fluency test* (Carlesimo et al., 1996) and the *Modified Card Sorting Test* (MCST, Caffarra et al., 2004). Finally, to evaluate general cognitive abilities we used the *Colored Progressive Matrices* (CPM; Carlesimo et al., 1996) and the *Clock Drawing Test* (CDT; Mondini et al., 2011).

### Procedure

Controls were tested in their own home in the area of Trento (Italy), whereas PD participants were tested at CeRiN, Trento (Italy). During the tasks, participants were seated at a distance of approximately 60 cm in front of a 15-inch PC monitor screen. E-Prime^®^2.0 (Schneider et al., 2002) was used to program and run the experiments. PD participants were tested during one experimental session that lasted approximately 60 min. Neuropsychological information of PD participants was collected from clinical records. Controls were tested in two separate experimental sessions lasting approximately 60 min each for completion of the neuropsychological assessment and experimental tasks, respectively. Written informed consent was collected from all the participants and the study was conducted in accordance with Helsinki Declaration (59th WMA General Assembly, Seoul, 2008). The study was approved by the ethic committee of the Department of General Psychology and the CeRiN—CIMec ethical committee.

### Statistical Analyses

For the time bisection task, for each participant a 7-point psychometric function was traced, plotting the seven comparison intervals on the x-axis and the probability of responding “long” on the y-axis. The cumulative normal function was fitted to the resulting curves. We calculated two indices, one that defines the perceived duration and one for sensitivity. The first was the Point of Subjective Equality (PSE), that is, the stimulus duration at which participants responded “short” or “long” with equal frequency. An observed shift of the bisection point can be interpreted as an indicator of differences in perceived duration, with smaller bisection point values meaning longer perceived durations. The second dependent variable was the Weber ratio (WR), which is based on one standard deviation (SD) on the psychometric function. The WR is the SD divided by the midpoint duration used in the experiment. This is a measure of temporal sensitivity; smaller values indicate more sensitive timing (Mioni et al., 2016). Separate *t*-tests were conducted on PSE and WR and we estimated effect size with Cohen’s *d*. One PD participant was excluded from the analyses because above 3 *SD* from their individual task mean condition. Therefore, for the time bisection task, the analyses were conducted on 39 participants (19 PD participants and 20 controls).

For the foreperiod task, data from practice trials, the first trial in each block, trials with premature responses (i.e., responses before target onset, 5.02% of the remaining trials for PD and 3.87% for controls), trials with RT below 150 ms (0.43% of the remaining trials for PD and 0.79% for controls) and trials without responses (0.64% of the remaining trials for PD and 0% for controls) were rejected from the analysis. Additionally, for each participant, trials with an RT above 3 *SD* from their individual task mean condition were treated as outliers and discarded from the RT analysis (1.45% of the remaining trials for PD and 1.8% for controls).

Mean RTs for each participant and condition were analyzed through a three-way mixed factorial analysis of variance (ANOVA) with *Group* (PD, controls) as a between-subjects factor, and *Type of block* (fixed, variable) and *Foreperiod* (1000 ms, 3000 ms) as within-subjects factors. Sequential effects were analyzed on the variable foreperiod trials only with a three-way mixed factorial ANOVA involving *Group* (PD, controls) as a between-subjects factor and *Foreperiod of the previous trial* (1000 ms, 3000 ms) and *Foreperiod of the current trial* (1000 ms, 3000 ms) as within-subject factors. One PD patient and one participant from the control group were excluded as they had less than 50% of correct trials in some task conditions. Moreover, two PD participants and one participant from the control group did not complete the task. Therefore, for the foreperiod task, the analyses were conducted on 35 participants (17 PD participants and 18 controls). All significant effects were followed by two-tailed paired *t*-tests and the effect size was estimated either with partial eta squared ($\eta_{\text{p}}^{2}$) or Cohen’s *d*.

Separate *t*-tests were conducted on neuropsychological tasks between PD participants and controls.

## Results

### Explicit Timing: Time Bisection Task

**Figure 2:**
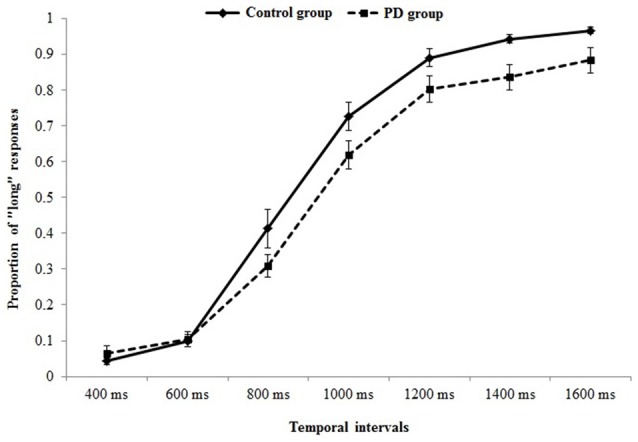
Psychometric function indicating the probability of “long” responses, for each comparison interval in Parkinson’s disease (PD) participants and controls.

Figure 2 represents the probability of “long” responses, for each comparison interval in PD participants and controls.

**Figure 3:**
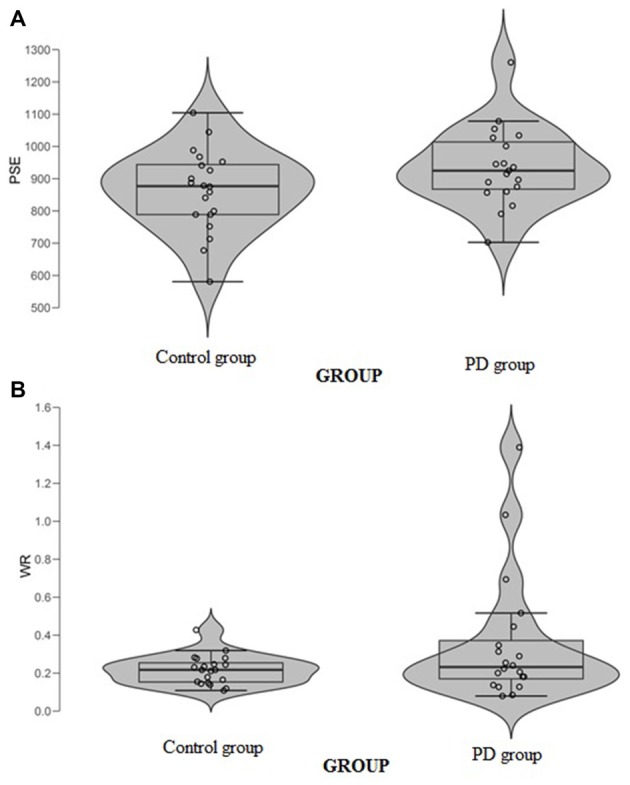
**(A)** Point of subjective equality (PSE) and **(B)** Weber Ratio (WR) of the time bisection task as a function of groups. Each dot represents a single participant.

When data were analyzed in term of PSE, a significant main effect of *Group* (*t*_(37)_ = 2.32, *p* = 0.026, Cohen’s *d* = 0.74) was found (Figure 3A); the PD participants’ PSE was shifted through the right indicating shorter perceived durations (PD participants PSE = 956, *SD* = 123; controls PSE = 863, *SD* = 126). When data were analyzed in term of WR, a significant main effect of *Group* (*t*_(37)_ = 2.03, *p* = 0.049, Cohen’s *d* = 0.64) was also found (Figure 3B); the PD participants’ WR was higher than the controls’ one, indicating lower temporal sensitivity (PD participants WR = 0.33, *SD* = 0.23; controls WR = 0.22, *SD* = 0.07).

### Implicit Timing: Foreperiod Task

#### Foreperiod Effects

**Figure 4:**
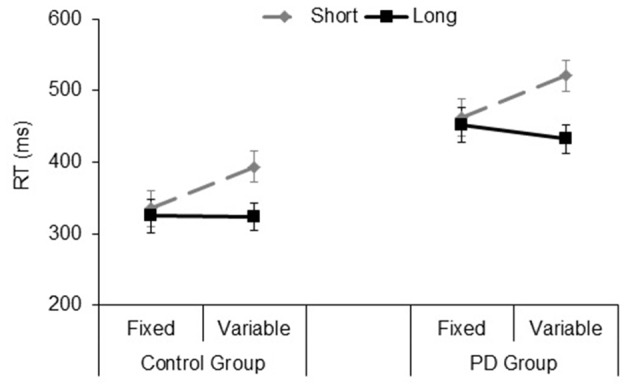
Mean reaction time (RT) plotted as a function of Type of Block (fixed, variable) and Foreperiod (short, long) in the Control group and PD group. Error bars represent the standard error of the mean.

The significant main effect of *Group* (*F*_(1,33)_ = 16.9, *p* < 0.001, $\eta_{\text{p}}^{2}$ = 0.33), showed that PD participants were slower than their controls (mean RT: 467 and 344 ms, respectively). The significant *Type of block* (*F*_(1,33)_ = 9.2, *p* = 0.005, $\eta_{\text{p}}^{2}$ = 0.21), and *Foreperiod* (*F*_(1,33)_ = 38.6, *p* < 0.001, $\eta_{\text{p}}^{2}$ = 0.53) main effects revealed faster responses in the fixed foreperiod paradigm compared to the variable one, and in the long foreperiod compared to the short foreperiod, respectively. Further, there was a significant interaction between *Type of block* and *Foreperiod* factors (*F*_(1,33)_ = 36.7, *p* < 0.001, $\eta_{\text{p}}^{2}$ = 0.52). This interaction was explained by the fact that participants were faster after the long foreperiod compared to the short foreperiod in the variable foreperiod paradigm (*t*_(34)_ *=* 8.97, *p* < 0.001, Cohen’s *d* = 1.56), while there was no difference between the two foreperiods in the fixed paradigm (*t*_(34)_ *=* 1.1, *p* = 0.27, Cohen’s *d* = 0.19). Hence, this result reflects the presence of the typical variable foreperiod effect and the absence of the fixed foreperiod one. Inspection of the data (see Figure 4) also showed that participants were faster at the short foreperiod when it was kept fixed across the block as compared to when it was intermixed across trials with the long foreperiod (*t*_(34)_ *=* 5.67, *p* < 0.001, Cohen’s *d* = 0.98). By contrast, participants were equally fast at the long foreperiod in both Types of blocks (*t*_(34)_ *=* 1.09, *p =* 0.28, Cohen’s *d* = 0.20). There were no significant interactions involving *Group* (all *p*s > 0.40). Please note that results remained the same even after logarithmic transformation of raw RT data, which controls for the difference in speed between the two groups (e.g., Ben-David et al., 2014).

#### Sequential Effects

**Figure 5:**
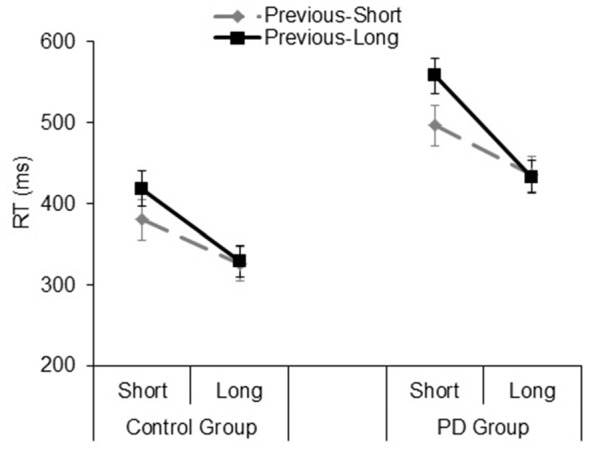
Mean reaction time (RT) plotted as a function of Previous Foreperiod (previous-short, previous-long) and Current Foreperiod (short, long) in the Control group and PD group. Error bars represent the standard error of the mean.

The analysis on the sequential effects showed significant main effects of the *Foreperiod of the previous trial* (*F*_(1,33)_ = 17.04, *p* = 0.001, $\eta_{\text{p}}^{2}$ = 0.34) and *Foreperiod of the current trial* (*F*_(1,33)_ = 64.66, *p* < 0.001, $\eta_{\text{p}}^{2}$ = 0.66), which were further explained by a significant interaction involving these two factors (*F*_(1,33)_ = 24.56, *p* < 0.001, $\eta_{\text{p}}^{2}$ = 0.42). This interaction reflected the typical pattern of asymmetrical sequential effects, that is, faster responses for the current short foreperiod when it was preceded by another short rather than long foreperiod (*t*_(34)_ *=* 5.15, *p* < 0.001, Cohen’s *d* = 0.87), while equally fast responses were observed for the current long foreperiod irrespective of the type of foreperiod occurring in the previous trial (*t*_(34)_ = 0.05, *p* = 0.95, Cohen’s *d* = 0.009). Apart from a significant *Group* main effect (*F*_(1,33)_ = 17.26, *p* < 0.001, $\eta_{\text{p}}^{2}$ = 0.34), the interactions involving the group factor were not significant (all *p*s > 0.14; Figure 5). As for the analysis on the foreperiod effects, all the results concerning sequential effects were replicated using log-transformed RT data.

#### Neuropsychological Evaluation

*T*-test analyses were conducted to test performance on neuropsychological tests in PD participants and controls (Table 1). Significant differences were observed between groups on TMT part-B (*p* = 0.050) and TMT B–A (*p* = 0.014), attentional matrices (*p* = 0.001) and CPM (*p* = 0.029) indicating that PD participants had lower attentional, visual search and non-verbal intelligence. No differences between groups were observed on the other measures (all *p*s > 0.05).

**Figure 6:**
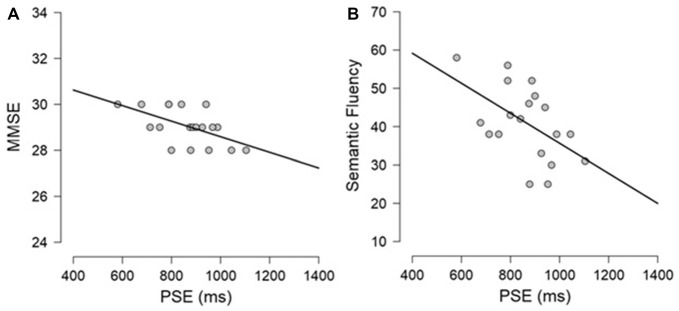
Graphical representation of the Pearson’s correlational analyses conducted between **(A)** PSE and Mini-Mental State Examination (MMSE) and **(B)** PSE and Semantic fluency in the control group.

**Figure 7:**
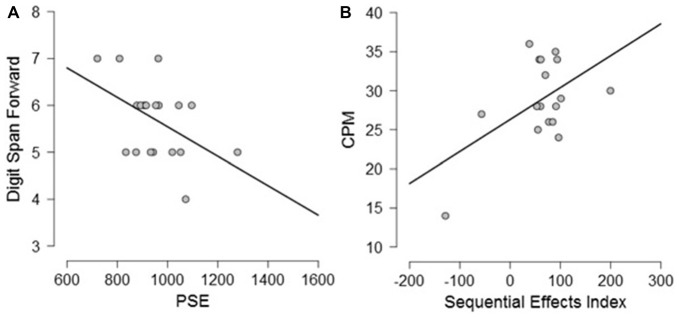
Graphical representation of the correlational analyses conducted between **(A)** PSE and Digit Span forwards and **(B)** between Sequential Effects Index and Color Progressive Matrix (CPM) in PD participants.

Exploratory Pearson correlational analyses were conducted between performance on the neuropsychological tasks and performance on the timing tasks separately for PD participants and controls. As measure of explicit timing we used the PSE, whereas for implicit timing we calculated a variable foreperiod effect index (short foreperiod minus long foreperiod RTs) and a sequential effects index (previous long minus previous short foreperiod RTs for current short foreperiod trials; for similar measures, see Triviño et al., 2011, 2016). Previous studies have suggested an involvement of attention, working memory and executive functions in explicit timing (Perbal et al., 2005; Aarsland et al., 2010; Parker et al., 2013b). Despite the small sample size prevents us from drawing clear conclusions regarding the relationship between cognitive functions and processing of explicit and implicit time, the following correlations emerged. Briefly, within the control group, negative correlations were observed between explicit timing and MMSE (*r* = −0.563, *p* = 0.012) and Semantic fluency (*r* = −0.525, *p* = 0.021) suggesting that participants with a lower MMSE score and a lower score at semantic fluency underestimated more in the explicit timing task (see Figure 6). No significant correlations were observed between implicit timing and any of the measures included in the neuropsychological evaluation (all *r* ≤ 0.23, all *p* ≥ 0.05). Within the PD group, a negative correlation was observed between explicit timing and Digit span forwards (*r* = −0.471, *p* = 0.042) suggesting that PD participants who had lower span underestimated time intervals in explicit timing tasks. Moreover, a negative correlation was observed in the implicit timing between the sequential effects index and CPM (*r* = −0.526, *p* = 0.030) indicating that PD participants who scored higher on the CPM test had smaller sequential effects (see Figure 7).

## Discussion

While there have been several studies investigating either explicit or implicit timing in PD, to our knowledge, only few of them have compared both explicit and implicit processes within a single experimental session (Jones et al., 2008; de Hemptinne et al., 2013). Overall, our results showed that impaired processing of explicit timing in PD could occur in the presence of spared implicit timing.

Specifically, regarding *explicit timing*, our results are in line with some previous studies (Smith et al., 2007; Mioni et al., 2016, 2017) showing higher PSE and higher WR in PD participants than in controls, indicating temporal under-estimation and lower temporal sensitivity in PD participants as compared to controls. Differently, Wearden et al. (2008) found no evidence of temporal impairment in PD participants within the sub-second range (100–800 ms) regardless of whether they were tested “on” or “off” medication. Moreover, Merchant et al. (2008) only reported higher temporal variability in PD participants compared to controls when tested with brief temporal intervals (350–1000 ms) but no differences in the perceived duration. These results occurred just when PD participants were tested “off” medication.

Some methodological differences might explain the different results with respect to our study. Merchant et al. (2008) and Wearden et al. (2008) used auditory stimuli and the superiority of audition over vision for temporal processing is well known. Sensitivity to time is much higher (lower threshold, or less variability) when intervals are marked by auditory rather than by visual signals (for a review, see Grondin, 2003). The underestimation observed in Smith et al. (2007) was restricted to long temporal intervals (1–5 s) while participants equally judged short temporal intervals (100–500 ms). Clinical characteristics were similar in Smith’s study and ours and in both studies PD participants were tested “on” medication. Moreover, we used visually filled intervals whereas Smith’s study included visually empty intervals. Previous studies showed that for brief temporal intervals (<300 ms), time discrimination was better with empty intervals in both visual and auditory modalities (Grondin, 1993). It is, then, possible that both PD and controls benefited from the presentation of empty intervals. The two studies reported by Mioni et al. (2016, 2017) showed under-estimation in PD participants with mild cognitive impairment (MCI) indicating that part of the temporal impairment observed in PD participants was explained by reduced cognitive abilities. In the present study, we were not able to differentiate the sample on the basis of diagnosis of MCI.

More consistent across different studies are the results regarding the WR. Here we observed higher variability in PD participants compared to controls. Merchant et al. (2008) also showed higher temporal variability in PD participants compared to controls when tested with brief temporal intervals (350–1000 ms), and Smith et al. (2007) showed higher variability across modalities (visual vs. auditory) and temporal ranges (1–5 s). Higher temporal variability in PD participants is often observed on tasks requiring motor responses (Jones and Jahanshahi, 2014). Interestingly, here we used a timing task (time bisection) that has a minimal motor component (Kopec and Brody, 2010; Gil and Droit-Volet, 2011), and we analyzed temporal performance excluding measures of reaction times that might have been affected by PD participants’ motor dysfunction (Jones and Jahanshahi, 2014).

Regarding *implicit timing*, participants with PD performed at the same level as controls in the variable foreperiod task, thus displaying the typical pattern of foreperiod and sequential effects. On the one hand, these findings are in line with previous observations that the variable foreperiod effect is generally preserved in PD (e.g., Bloxham et al., 1987; Jahanshahi et al., 1992), at least when using relatively short durations for the longest foreperiod (~3 s; see Jurkowski et al., 2005). On the other hand, our findings extend previous research by also revealing unimpaired sequential effects in participants with PD. The presence of sequential effects in PD suggests that such effects are not related to dopamine-dependent neural mechanisms.

An unexpected result concerning implicit timing was the lack of the fixed foreperiod effect in both PD and control groups. In contrast to the literature showing a RT advantage on the fixed short blocks compared to the longest ones, here there was no difference between short and long blocks. It is interesting to note, however, that performance on short foreperiods significantly changed as a function of the task context (fixed vs. variable), although not as strongly to get the typical fixed foreperiod effect. This result thus suggests that there was still a difference in the processing of the short foreperiods between the fixed and variable foreperiod tasks.

Beyond PD, similar dissociations between explicit and implicit timing have been also documented in other types of participants and kinds of tasks. For instance, Bégel et al. (2017) recently showed that individuals with “beat deafness”, a congenital anomaly associated with difficulties in synchronizing to the beat, performed poorly on explicit rhythm but not on implicit rhythm tasks. Likewise, it has been shown that participants with right frontal damage have troubles in orienting attention to time intervals when they are cued by explicit information but not when using implicit rhythmic patterns (Triviño et al., 2010, 2011). Further supporting the idea that explicit and implicit timing reflect distinct processes, it seems that the two follow distinct developmental trajectories being the explicit aspect more variable across age groups compared to the implicit one (e.g., Droit-Volet and Coull, 2016; but also see Mento and Tarantino, 2015). Finally, explicit and implicit timing have been related to distinct neural regions. Specifically, explicit timing is usually associated with the supplementary motor area, basal ganglia, cerebellum and right inferior frontal and parietal cortices (Coull and Nobre, 2008; Wiener et al., 2010). Implicit timing, when measured through the variable foreperiod effect, has been linked to the functioning of the right lateral prefrontal cortex (Arbula et al., 2017) and at least in one occurrence also of the left one (Triviño et al., 2010), whereas sequential effects have been related to the motor/premotor circuitry (Vallesi et al., 2007a) and left subcortical structures (Triviño et al., 2016).

Taken together, our results add to the neural dissociation between explicit and implicit timing by demonstrating that accurate performance in the time bisection task, but not in the foreperiod task, depends on intact basal ganglia and dopaminergic functions. This is in line with several data from rodents showing that manipulation of dopamine in the substantia nigra changes the perception of time (Meck, 2006; Soares et al., 2016). It has been recently proposed that medial frontal dopamine, which can degenerate in PD (Parker et al., 2015a; Kim et al., 2017), is also critical for accurate timing behavior in rodents (Parker et al., 2015b). Interestingly, depletion of dopamine input from the ventral tegmental area to the medial prefrontal cortex disrupts the foreperiod effect of rats engaged in a simple reaction time task (Parker et al., 2013a). This result is thus at odds with both our findings and others’ ones showing a normal foreperiod effect in participants with PD (Bloxham et al., 1987; Jahanshahi et al., 1992). As suggested by the authors, however, it might be possible that only those patients with executive dysfunctions have impaired prefrontal dopamine regulation, which would explain the discrepancy between animal and human findings reported in the context of foreperiod tasks. Indeed, our participants with PD did not have a severe executive dysfunction as shown by the neuropsychological evaluation.

In keeping with the contribution of cognitive factors on temporal processing, despite the evidence of reduced cognitive abilities in PD, very few studies have identified cognitive dysfunction in temporal processing in PD participants (Jones and Jahanshahi, 2014). One interesting exception is Perbal et al. (2005), who used time production and reproduction tasks as well as neuropsychological measures with PD participants and controls. Correlations conducted on the entire sample showed that participants with higher temporal variability in the time reproduction and time production tasks had lower short-term memory and working memory abilities. Among the studies that used similar explicit and implicit timing tasks, Mioni et al. (2016, 2017) showed greater under-estimation (higher PSE) and higher variability (WR) in PD participants with MCI, confirming that cognitive factors can influence performance on explicit time processing, which aligns with the documented cognitive deficits of this group (see also Merchant et al., 2008, for different findings). The results obtained from the preliminary correlations conducted in our study are in line with previous findings indicating that participants with lower cognitive abilities produced a greater underestimation (in the explicit timing task). Only in PD participants negative correlations were observed in implicit timing between the sequential effects index and CPM, indicating that PD participants who scored higher on the CPM test had smaller sequential effects. According to this exploratory correlation, it seems that PD participants with greater cognitive abilities were less influenced by the foreperiod duration provided by the previous trial. This might imply more focus on the current trial temporal information and greater resistance to lower-level influences from previous trial durations. However, since the correlation for the implicit task represents a novel finding, caution has to be taken before drawing firm conclusions on the role of general cognitive abilities in the expression of sequential effects.

Among the limitations of the present study, it is important to acknowledge the quite small sample size and the difference in the temporal ranges used for the explicit and implicit timing tasks. Regarding the former point, our sample size is comparable to sample sizes used in previous work with PD participants (Jones and Jahanshahi, 2014). Moreover, the differences found between the two groups can still provide interesting insights into the understanding of the different processes underlying explicit and implicit timing. Regarding the latter point, it would be highly informative in future studies to match the durations between explicit and implicit timing. For the explicit part of our design, we opted for these range of durations (400–1600 ms) to reduce the use of counting strategies that are often engaged when longer intervals (<1 s) are processed (Grondin, 2010). Conversely, for the implicit part, we decided to employ longer durations to take into account the motor deficit associated with PD participants (Jones and Jahanshahi, 2014). Despite these limitations, however, our work provides useful evidence on the dissociation between explicit and implicit timing in clinical populations. Future research should further explore such a dissociation by employing other measures of implicit and explicit timing with reduced motor component and by adopting the same temporal range in the two types of tasks.

To conclude, our results support the existence of two different processes underlying explicit and implicit timing in PD participants. Moreover, we extend previous studies in the field of implicit timing by providing the first experimental evidence of preserved sequential effects in PD.

## Author Contributions

All the authors were involved in the conception of the work. GM and MC are co-first authors, performed data analysis, drafted the manuscript and were involved in all subsequent revisions. GM and RDG were involved in data collection. AV, ÁC, RDG and FS provided ongoing contributions and feedback throughout the experimental process. They also provided additional revisions to the manuscript. All the authors have approved the final version of the manuscript and agree to be accountable for all aspects of the work.

## Conflict of Interest Statement

The authors declare that the research was conducted in the absence of any commercial or financial relationships that could be construed as a potential conflict of interest.

## Footnotes

^1^A complete description of the neurophysiological tasks used can be found in Mioni et al. (2016, 2017).

## References

[B1] Aarsland D. Bronnick K. Williams-Gray C. Weintraub D. Marder K. Kulisevsky J.. (2010). Mild cognitive impairment in Parkinson disease: a multicenter pooled analysis. Neurology 75, 1062–1069. 10.1212/wnl.0b013e3181f39d0e 20855849 PMC2942065

[B2] Alberico S. L. Kim Y. Lence T. Narayanan N. S. (2017). Axial levodopa-induced dyskinesias and neuronal activity in the dorsal striatum. Neuroscience 343, 240–249. 10.1016/j.neuroscience.2016.11.046 27956068 PMC5262537

[B3] Arbula S. Pacella V. De Pellegrin S. Rossetto M. Denaro L. D’Avella D.. (2017). Addressing the selective role of distinct prefrontal areas in response suppression: a study with brain tumor patients. Neuropsychologia 100, 120–130. 10.1016/j.neuropsychologia.2017.04.018 28412512 PMC5813715

[B4] Artieda J. Pastor M. A. Lacruz F. Obeso J. A. (1992). Temporal discrimination is abnormal in Parkinson’s disease. Brain 115, 199–210. 10.1093/brain/115.1.199 1559154

[B5] Bausenhart K. M. Rolke B. Ulrich R. (2008). Temporal preparation improves temporal resolution: evidence from constant foreperiods. Percept. Psychophys. 70, 1504–1514. 10.3758/pp.70.8.1504 19064493

[B103] Bégel V. Benoit C. E. Correa A. Cutanda D. Kotz S. A. Dalla Bella S. (2017). “Lost in time” but still moving to the beat. Neuropsychologia 94, 129–138. 10.1016/j.neuropsychologia.2016.11.022 27914979

[B6] Ben-David B. M. Eidels A. Donkin C. (2014). Effects of aging and distractors on detection of redundant visual targets and capacity: do older adults integrate visual targets differently than younger adults? PLoS One 9:e113551. 10.1371/journal.pone.0113551 25501850 PMC4264737

[B7] Bloxham C. A. Dick D. J. Moore M. (1987). Reaction times and attention in Parkinson’s disease. J. Neurol. Neurosurg. Psychiatry 50, 1178–1183. 10.1136/jnnp.50.9.1178 3668566 PMC1032352

[B8] Brown V. J. Robbins T. W. (1991). Simple and choice reaction time performance following unilateral striatal dopamine depletion in the rat. Impaired motor readiness but preserved response preparation. Brain 114, 513–525. 10.1093/brain/114.1.513 2004254

[B9] Caffarra P. Vezzadini G. Dieci F. Zonato F. Venneri A. (2004). Modified card sorting test: normative data. J. Clin. Exp. Neuropsychol. 26, 246–250. 10.1076/jcen.26.2.246.28087 15202543

[B10] Calabresi P. Picconi B. Parnetti L. Di Filippo M. (2006). A convergent model for cognitive dysfunctions in Parkinson’s disease: the critical dopamine-acetylcholine synaptic balance. Lancet Neurol. 5, 974–983. 10.1016/S1474-4422(06)70600-7 17052664

[B11] Capizzi M. Correa Á. Wojtowicz A. Rafal R. D. (2015). Foreperiod priming in temporal preparation: testing current models of sequential effects. Cognition 134, 39–49. 10.1016/j.cognition.2014.09.002 25460377

[B12] Carlesimo G. A. Caltagirone C. Gainotti G. Fadda L. Gallassi R. Lorusso S.. (1996). The Mental deterioration battery: normative data, diagnostic reliability and qualitative analyses of cognitive impairment. Eur. Neurol. 36, 378–384. 10.1159/000117297 8954307

[B14] Coull J. (2009). Neural substrates of mounting temporal expectation. PLoS Biol. 7:e1000166. 10.1371/journal.pbio.1000166 19652699 PMC2711332

[B15] Coull J. T. Hwang H. J. Leyton M. Daghter A. (2012). Dopamine precursor depletion impairs timing in healthy volunteers by attenuating activity in putamen and supplementary motor area. J. Neurosci. 32, 16704–16715. 10.1523/jneurosci.1258-12.2012 23175824 PMC6621775

[B17] Coull J. T. Nobre A. C. (2008). Dissociating explicit timing from temporal expectation with fMRI. Curr. Opin. Neurobiol. 18, 137–144. 10.1016/j.conb.2008.07.011 18692573

[B18] de Hemptinne C. Ivanoiu A. Lefèvre P. Missal M. (2013). How does Parkinson’s disease and aging affect temporal expectation and the implicit timing of eye movements? Neuropsychologia 51, 340–348. 10.1016/j.neuropsychologia.2012.10.001 23063965

[B102] Droit-Volet S. Coull J. T. (2016). Distinct developmental trajectories for explicit and implicit timing. J. Exp. Child Psychol. 150, 141–154. 10.1016/j.jecp.2016.05.010 27295205

[B19] Dubois B. Burn D. Goetz C. Aarsland D. Brown R. G. Broe G. A.. (2007). Diagnostic procedures for Parkinson’s disease dementia: recommendations from the movement disorders society task force. Mov. Disord. 22, 2314–2324. 10.1002/mds.21844 18098298

[B20] Folstein M. F. Folstein S. E. McHugh P. R. (1975). “Mini-mental state”. A practical method for grading the cognitive state of participants for the clinician. J. Psychiatr. Res. 12, 189–198. 10.1016/0022-3956(75)90026-6 1202204

[B21] Gibbon J. (1977). Scalar expectancy theory and Weber’s law in animal timing. Psychol. Rev. 84, 279–325. 10.1037//0033-295x.84.3.279

[B22] Gil S. Droit-Volet S. (2011). “Time flies in the presence of angry faces”… depending on the temporal task used!. Acta Psychol. 136, 354–362. 10.1016/j.actpsy.2010.12.010 21276583

[B23] Giovagnoli A. R. Del Pesce M. Mascheroni S. Simoncelli M. Laiacona M. Capitani E. (1996). Trail making test: normative values from 287 normal adult controls. Ital. J. Neurol. Sci. 17, 305–309. 10.1007/bf01997792 8915764

[B24] Goetz C. G. Poewe W. Rascol O. Sampaio C. Stebbins G. T. Counsell C.. (2004). Movement Disorder Society Task Force report on the Hoehn and Yahr staging scale: status and recommendations. Mov. Disord. 19, 1020–1028. 10.1002/mds.20213 15372591

[B25] Grondin S. (1993). Duration discrimination of empty and filled intervals marked by auditory and visual signals. Percept. Psychophys. 54, 383–394. 10.3758/bf03205274 8414897

[B26] Grondin S. (2003). “Sensory modalities and temporal processing,” in Time and Mind II, ed. Helfrich H. (Göttingen: Hogrefe and Huber), 61–77.

[B27] Grondin S. (2010). Timing and time perception: a review of recent behavioral and neuroscience findings and theoretical directions. Atten. Percept. Psychophys. 72, 561–582. 10.3758/app.72.3.561 20348562

[B28] Jahanshahi M. Brown B. G. Marsden C. D. (1992). The effect of withdrawal of dopaminergic medication on simple and choice reaction time and the use of advance information in Parkinson’s disease. J. Neurol. Neurosurg. Psychiatry 55, 1168–1176. 10.1136/jnnp.55.12.1168 1362212 PMC1015334

[B29] Jones C. R. G. Jahanshahi M. (2014). “Motor and perceptual timing in Parkinson’s disease,” in Neurobiology of Interval Timing. Advances in Experimental Medicine and Biology, eds Merchant H. de Lafuente V. (New York, NY: Springer Verlag), 265–290. 10.1007/978-1-4939-1782-2_14 25358715

[B30] Jones C. R. G. Malone T. J. L. Dirnberger G. Edwards M. Jahanshahi M. (2008). Basal ganglia, dopamine and temporal processing: performance on three timing tasks on and off medication in Parkinson’s disease. Brain Cogn. 68, 30–41. 10.1016/j.bandc.2008.02.121 18378374

[B31] Jurkowski A. J. Stepp E. Hackley S. A. (2005). Variable foreperiod deficits in Parkinson’s disease: dissociation across reflexive and voluntary behaviors. Brain Cogn. 58, 49–61. 10.1016/j.bandc.2004.09.008 15878726

[B32] Kim Y. Han S. Alberico S. L. Ruggiero R. N. De Corte B. Chen K.. (2017). Optogenetic stimulation of frontal D1 neurons compensates for impaired temporal contol of action in dopamine-depleted mice. Curr. Biol. 27, 39–47. 10.1016/j.cub.2016.11.029 27989675 PMC5225083

[B33] Koch G. Costa A. Brusa L. Peppe A. Gatto I. Torriero S.. (2008). Impaired reproduction of second but not millisecond time intervals in Parkinson’s disease. Neuropsychologia 46, 1305–1313. 10.1016/j.neuropsychologia.2007.12.005 18215403

[B34] Kopec C. D. Brody C. D. (2010). Human performance on the temporal bisection task. Brain Cogn. 74, 262–272. 10.1016/j.bandc.2010.08.006 20846774 PMC3034315

[B35] Kudlicka A. Clare L. Hindle J. V. (2011). Executive functions in Parkinson’s disease: systematic review and meta-analysis. Mov. Disord. 26, 2305–2315. 10.1002/mds.23868 21971697

[B36] Lange K. W. Tucha O. Steup A. Gsell W. Naumann M. (1995). Subjective time estimation in Parkinson’s disease. J. Neural Transm. Suppl. 46, 433–438. 8821079

[B37] Lee E.-Y. Valle-Inclán F. Hackley S. A. (2012). Decomposition of warning effects in Parkinson’s disease. Neuropsychol. Dev. Cogn. B Aging Neuropsychol. Cogn. 19, 433–447. 10.1080/13825585.2011.630717 22149180

[B39] Los S. A. Kruijne W. Meeter M. (2014). Outlines of a multiple trace theory of temporal preparation. Front. Psychol. 5:1058. 10.3389/fpsyg.2014.01058 25285088 PMC4168672

[B38] Los S. A. van den Heuvel C. E. (2001). Intentional and unintentional contributions to nonspecific preparation during reaction time foreperiods. J. Exp. Psychol. Hum. Percept. Perform. 27, 370–386. 10.1037/0096-1523.27.2.370 11318053

[B40] Malapani C. Rakitin B. Levy R. Meck W. H. Deweer B. Dubois B.. (1998). Coupled temporal memories in Parkinson’s disease: a dopamine-related dysfunction. J. Cogn. Neurosci. 10, 316–331. 10.1162/089892998562762 9869707

[B41] Marinho V. Oliveira T. Rocha K. Ribeiro J. Magalhães F. Bento T.. (2018). The dopaminergic system dynamic in the time perception: a review of the evidence. Int. J. Neurosci. 128, 262–282. 10.1080/00207454.2017.1385614 28950734

[B42] Mattes S. Ulrich R. (1997). Response force is sensitive to the temporal uncertainty of response stimuli. Percept. Psychophys. 59, 1089–1097. 10.3758/bf03205523 9360481

[B43] Meck W. H. (2006). Neuroanatomical localization of an internal clock: a functional link between mesolimbic, nigrostriatal, and mesocortical dopaminergic systems. Brain Res. 1109, 93–107. 10.1016/j.brainres.2006.06.031 16890210

[B44] Meck W. H. Penney T. B. Pouthas V. (2008). Cortico-striatal representation of time in animals and humans. Curr. Opin. Neurobiol. 18, 145–152. 10.1016/j.conb.2008.08.002 18708142

[B45] Mento G. (2017). The role of the P3 and CNV components in voluntary and automatic temporal orienting: a high spatial-resolution ERP study. Neuropsychologia 107, 31–40. 10.1016/j.neuropsychologia.2017.10.037 29109036

[B46] Mento G. Tarantino V. (2015). Developmental trajectories of internally and externally driven temporal prediction. PLoS One 10:e0135098. 10.1371/journal.pone.0135098 26262878 PMC4532408

[B47] Mento G. Tarantino V. Vallesi A. Bisiacchi P. S. (2015). Spatiotemporal neurodynamics underlying internally and externally driven temporal prediction: a high spatial resolution ERP study. J. Cogn. Neurosci. 27, 425–439. 10.1162/jocn_a_00715 25203276

[B48] Merchant H. Luciana M. Hooper C. Majestic S. Tuite P. (2008). Interval timing and Parkinson’s disease: heterogeneity in temporal performance. Exp. Brain Res. 184, 233–248. 10.1007/s00221-007-1097-7 17828600

[B49] Mioni G. Grondin S. Meligrana L. Perini F. Bartolomei L. Stablum F. (2017). Effects of happy and sad facial expressions on the perception of time in Parkinson’s disease participants with mild cognitive impairment. J. Clin. Exp. Neuropsychol. 10.1080/13803395.2017.1324021 28532288

[B50] Mioni G. Meligrana L. Grondin S. Perini F. Bartolomei L. Stablum F. (2016). Effects of emotional facial expression on time perception in participants with Parkinson’s disease. J. Int. Neuropsychol. Soc. 22, 890–899. 10.1017/S1355617715000612 26250885

[B51] Mondini S. Mapelli D. Vestri A. Arcara G. Bisiacchi P. S. (2011). Esame Neuropsicologico Breve 2. Una Batteria di Test Per lo Screening Neuropsicologico. Milano: Raffaello Cortina.

[B52] Movement Disorder Society Task Force on Rating Scales for Parkinson’s Disease. (2003). The unified Parkinson’s disease rating scale (UPDRS): status and recommendations. Mov. Disord. 18, 738–750. 10.1002/mds.10473 12815652

[B53] Nalls M. A. McLean C. Y. Rick J. Eberly S. Hutten S. J. Gwinn K.. (2015). Diagnosis of Parkinson’s disease on the basis of clinical and genetic classification: a population-based modelling study. Lancet Neurol. 14, 1002–1009. 10.1016/S1474-4422(15)00178-7 26271532 PMC4575273

[B54] Niemi P. Näätänen R. (1981). Foreperiod and reaction time. Psychol. Bull. 89, 133–162. 10.1037/0033-2909.89.1.133

[B55] Novelli G. Papagno C. Capitani E. Laiacona M. Vallar G. Cappa S. F. (1986). Tre test clinici di ricerca e produzione lessicale: taratura su soggetti normali. Arch Psicol. Neurol. Psichiatr. 4, 477–506.

[B56] O’Boyle D. J. Freeman J. S. Cody F. W. (1996). The accuracy and precision of timing of self-paced, repetitive movements in subjects with Parkinson’s disease. Brain 119, 51–70. 10.1093/brain/119.1.51 8624694

[B58] Parker K. L. Alberico S. L. Miller A. D. Narayanan N. S. (2013a). Prefrontal D1 dopamine signaling is necessary for temporal expectation during reaction time performance. Neuroscience 255, 246–254. 10.1016/j.neuroscience.2013.09.057 24120554 PMC3856920

[B57] Parker K. L. Lamichhane D. Caetano M. S. Narayanan N. S. (2013b). Executive dysfunction in Parkinson’s disease and timing deficits. Front. Integr. Neurosci. 7:75. 10.3389/fnint.2013.00075 24198770 PMC3813949

[B59] Parker K. L. Chen K. H. Kingyon J. R. Cavanagh J. F. Narayanan N. S. (2015a). Medial frontal ~4-Hz activity in humans and rodents is attenuated in PD patients and in rodents with cortical dopamine depletion. J. Neurophysiol. 114, 1310–1320. 10.1152/jn.00412.2015 26133799 PMC4588517

[B60] Parker K. L. Ruggiero R. N. Narayanan N. S. (2015b). Infusion of D1 dopamine receptor agonist into medial frontal cortex disrupts neural correlates of interval timing. Front. Behav. Neurosci. 9:294. 10.3389/fnbeh.2015.00294 26617499 PMC4639709

[B61] Pastor M. A. Artieda J. Jahanshahi M. Obeso J. A. (1992a). Time estimation and reproduction is abnormal in Parkinson’s disease. Brain 115, 211–225. 10.1093/brain/115.1.211 1559155

[B62] Pastor M. A. Jahanshahi M. Artieda J. Obeso J. A. (1992b). Performance of repetitive wrist movements in Parkinson’s disease. Brain 115, 875–891. 10.1093/brain/115.3.875 1628206

[B63] Perbal S. Deweer B. Pillon B. Vidailhet M. Dubois B. Pouthas V. (2005). Effects of internal clock and memory disorders on duration reproductions and duration productions in participants with Parkinson’s disease. Brain Cogn. 58, 35–48. 10.1016/j.bandc.2005.02.003 15878725

[B65] Rafal R. D. Walker J. A. Posner M. I. Friedrich F. J. (1984). Cognition and the basal ganglia. Separating mental and motor components of performance in Parkinson’s disease. Brain 107, 1083–1094. 10.1093/brain/107.4.1083 6509309

[B66] Schneider W. Eschman A. Zuccolotto A. (2002). E-Prime User’s Guide. Pittsburgh: Psychology Software Tools Inc.

[B67] Smith J. G. Harper D. N. Gittings D. Abernethy D. (2007). The effect of Parkinson’s disease on time estimation as a function of stimulus duration range and modality. Brain Cogn. 64, 130–143. 10.1016/j.bandc.2007.01.005 17343966

[B68] Soares S. Atallah B. V. Paton J. J. (2016). Midbrain dopamine neurons control judgment of time. Science 354, 1273–1277. 10.1126/science.aah5234 27940870

[B69] Spinnler H. Tognoni G. (1987). Standardizzazione e taratura italiana di test neuropsicologici. Ital. J. Neurol. Sci. 8, 1–120. 3330072

[B70] Steinborn M. B. Rolke B. Bratzke D. Ulrich R. (2008). Sequential effects within a short foreperiod context: evidence for the conditioning account of temporal preparation. Acta Psychol. 129, 297–307. 10.1016/j.actpsy.2008.08.005 18804193

[B71] Triviño M. Arnedo M. Lupiáñez J. Chirivellac J. Correa A. (2011). Rhythms can overcome temporal orienting deficit after right frontal damage. Neuropsychologia 49, 3917–3930. 10.1016/j.neuropsychologia.2011.10.009 22019698

[B72] Triviño M. Correa Á. Arnedo M. Lupiáñez J. (2010). Temporal orienting deficit after prefrontal damage. Brain 133, 1173–1185. 10.1093/brain/awp346 20145048

[B73] Triviño M. Correa Á. Lupiáñez J. Funes M. J. Catena A. Humphreys G. W. (2016). Brain networks of temporal preparation: a multiple regression analysis of neuropsychological data. Neuroimage 142, 489–497. 10.1016/j.neuroimage.2016.08.017 27521744

[B75] Vallesi A. (2010). “Neuro-anatomical substrates of foreperiod effects,” in Attention and Time, eds Nobre A. Coull J. (Oxford: Oxford University Press), 303–316.

[B76] Vallesi A. Arbula S. Bernardis P. (2014). Functional dissociations in temporal preparation: evidence from dual-task performance. Cognition 130, 141–151. 10.1016/j.cognition.2013.10.006 24291265

[B100] Vallesi A. Lozano V. N. Correa A. (2013). Dissociating temporal preparation processes as a function of the inter-trial interval duration. Cognition 127, 22–30. 10.1016/j.cognition.2012.11.011 23318351

[B77] Vallesi A. McIntosh A. R. Shallice T. Stuss D. T. (2009). When time shapes behavior: fMRI evidence of brain correlates of temporal monitoring. J. Cogn. Neurosci. 21, 1116–1126. 10.1162/jocn.2009.21098 18752413

[B78] Vallesi A. Mussoni A. Mondani M. Budai R. Skrap M. Shallice T. (2007a). The neural basis of temporal preparation: insights from brain tumor participants. Neuropsychologia 45, 2755–2763. 10.1016/j.neuropsychologia.2007.04.017 17544014

[B79] Vallesi A. Shallice T. Walsh V. (2007b). Role of the prefrontal cortex in the foreperiod effect: TMS evidence for dual mechanisms in temporal preparation. Cereb. Cortex 17, 466–474. 10.1093/cercor/bhj163 16565293

[B74] Vallesi A. Shallice T. (2007). Developmental dissociations of preparation over time: deconstructing the variable foreperiod phenomena. J. Exp. Psychol. Hum. Percept. Perform. 33, 1377–1388. 10.1037/0096-1523.33.6.1377 18085950

[B80] Wearden J. H. Smith-Spark J. H. Cousins R. Edelstyn N. M. J. Cody F. W. J. O’Boyle D. J. (2008). Stimulus timing by people with Parkinson’s disease. Brain Cogn. 67, 264–279. 10.1016/j.bandc.2008.01.010 18329150

[B101] Wiener M. Turkeltaub P. E. Coslett H. B. (2010). Implicit timing activates the left inferior parietal cortex. Neuropsychologia 48, 3967–3971. 10.1016/j.neuropsychologia.2010.09.014 20863842 PMC2977988

[B81] Zahn T. P. Rosenthald D. Shakow D. (1963). Effects of irregular preparatory intervals on reaction time in schizophrenia. J. Abnorm. Soc. Psychol. 67, 44–52. 10.1037/h0049269 14003031

[B82] Zhang J. Nombela C. Wolpe N. Barker R. A. Rowe J. B. (2016). Time on timing: dissociating premature responding from interval sensitivity in Parkinson’s disease. Mov. Disord. 31, 1163–1172. 10.1002/mds.26631 27091513 PMC4988382

